# Sociodemographic Representativeness in a Nationwide Web-Based Survey of the View of Men on Involvement in Health Care Decision-Making: Cross-Sectional Questionnaire Study

**DOI:** 10.2196/19517

**Published:** 2020-09-02

**Authors:** Søren F Birkeland, Anders K Haakonsson, Susanne S Pedersen, Nina Rottmann, Michael J Barry, Sören Möller

**Affiliations:** 1 Open Patient Data Explorative Network (OPEN) Odense University Hospital and Department of Clinical Medicine University of Southern Denmark Odense Denmark; 2 Department of Psychology University of Southern Denmark Odense Denmark; 3 Department of Cardiology Odense University Hospital Odense Denmark; 4 REHPA, The Danish Knowledge Centre for Rehabilitation and Palliative Care Odense University Hospital and Department of Clinical Medicine University of Southern Denmark Nyborg Denmark; 5 MGH Division of General Internal Medicine Harvard Medical School Boston, MA United States

**Keywords:** research methodology, electronic data capture, internet-based survey, representativeness, generalizability, user involvement, patient satisfaction, bioethics, medical law, cancer

## Abstract

**Background:**

Being able to generalize research findings to a broader population outside of the study sample is an important goal in surveys on the internet. We conducted a nationwide, cross-sectional, web-based survey with vignettes illustrating different levels of patient involvement to investigate men’s preferences regarding participation in health care decision-making. Following randomization into vignette variants, we distributed the survey among men aged 45 to 70 years through the state-authorized digital mailbox provided by the Danish authorities for secure communication with citizens.

**Objective:**

This study aimed to investigate the sociodemographic representativeness of our sample of men obtained in a nationwide web-based survey using the digital mailbox.

**Methods:**

Response rate estimates were established, and comparisons were made between responders and nonresponders in terms of age profiles (eg, average age) and municipality-level information on sociodemographic characteristics.

**Results:**

Among 22,288 men invited during two waves, a total of 6756 (30.31%) participants responded to the survey. In adjusted analyses, responders’ characteristics mostly resembled those of nonresponders. Response rates, however, were significantly higher in older men (odds ratio [OR] 2.83 for responses among those aged 65-70 years compared with those aged 45-49 years, 95% CI 2.58-3.11; *P*<.001) and in rural areas (OR 1.10 compared with urban areas, 95% CI 1.03-1.18; *P*=.005). Furthermore, response rates appeared lower in areas with a higher tax base (OR 0.89 in the highest tertile, 95% CI 0.81-0.98; *P*=.02).

**Conclusions:**

Overall, the general population of men aged 45 to 70 years was represented very well by the responders to our web-based survey. However, the imbalances identified highlight the importance of supplementing survey findings with studies of the representativeness of other characteristics of the sample like trait and preference features, so that proper statistical corrections can be made in upcoming analyses of survey responses whenever needed.

## Introduction

Research has suggested that communication breaches are underlying issues in many complaints about health care delivery [1-3]. Beckman et al found that patients’ feelings of being deserted and poor information delivery were central themes in malpractice suits, and later research pointed in the same direction [2,3]. Insufficient patient involvement in decision-making has therefore been proposed as an important underlying issue when people file a malpractice complaint [4-6]. People’s lack of “ownership” of decisions about the care they have received may very well be an underlying cause in many complaints, and perhaps this is particularly the case if treatment leads to an undesirable outcome. Nonetheless, even if greater patient involvement seems to be an obvious starting point to increase patient satisfaction with health care and prevent complaints, there is still scant research to support this notion.

We therefore conducted a cross-sectional questionnaire study to increase our understanding of patient preferences for involvement in health care decision-making. The study was designed as a nationwide internet survey in the general population benefiting from the opportunities in Denmark for survey distribution through a web portal used for communication between authorities and citizens. As is the case with other survey approaches, however, using the internet for data collection may raise concerns about nonresponse bias and sample representativeness [7]. A representative survey sample can be defined as “one that has strong external validity in relationship to the target population the sample is meant to represent” [8]. This implies that results from the survey analyses can be generalized with confidence to the population of interest. Correspondingly, nonresponse and poor coverage of the sample may bias survey findings if the responding sample differs from the characteristics of the target population in nonnegligible ways [8]. For example, responders may more often be better off economically than the target population, thereby lowering the sample’s representativeness [8]. Similarly, response rates (RRs) may vary with age and location (eg, areas with various ethnic populations) [9]. In this paper, we report on the representativeness of our web-based survey in terms of *sociodemographic characteristics* through comparisons of our sample with nonresponders and national statistics data.

## Methods

### Setting and Participants

We used a web platform (REDCap [10]) for the survey and identified the sample with civil registration numbers through use of the Danish Health Data Authority (DHDA). Danish citizens are all registered in the civil registration system with unique personal identification numbers. We recruited participants using personal invitations delivered to the participants through coupling of civil registration numbers to the “digital mailbox” provided by the Danish authorities for safe communication with citizens. Adult Danish citizens are registered to use the digital mailbox as default, although a small proportion of citizens have actively deregistered (9.9% in 2017) [9]. Digital mailbox communication is encrypted, and thus, its security is higher than that of usual email and mails sent by regular postal service [9].

### Variables and Measurement

The survey illustrated various levels of patient involvement in health care decision-making through use of multiple case vignette versions. We used decision-making with regard to having a prostate-specific antigen (PSA) test for prostate cancer (PCa) screening as a model situation and measured responders’ imagined satisfaction with health care and readiness to initiate malpractice litigation about the health care received. Responders were randomized into one of 30 different scenarios with an identical core structure. There were differences regarding the degree of patient involvement (five levels), the decision to have a PSA test, and outcomes (three possibilities; details have been provided previously [11]). Measures comprised standardized validated instruments (eg, personality) and purpose-designed questions (eg, sociodemographic characteristics). Regarding participant age, we chose the age range of 45 to 70 years with reference to international guidelines about PCa screening [12,13]. During survey development, we collaborated with male adult health user representatives to optimize the survey’s content and acceptability.

We examined the overall representativeness of the sample obtained through the procedures described above. We estimated RRs and compared responders and nonresponders with respect to their age profile. Furthermore, we made comparisons regarding municipality-level sociodemographic characteristics derived from the 98 municipality codes available from the Danish municipality statistics database [14]. Data were used as standard measures for the state and municipalities in Denmark, as well as for research purposes, and included statistics information of municipality-level population density, tax per citizen figures, proportion of citizens with higher education, and proportion of citizens of non-Western origin [15,16]. Reporting in this article follows the STROBE (Strengthening the Reporting of Observational Studies in Epidemiology) guidelines for observational studies [17].

### Study Size, Quantitative Variables, and Statistical Methods

For the project, we drew a random sample of 12,000 male Danish health care users in the age range of 45 to 70 years, according to the following sample size estimation. When the primary outcome measure (readiness to complain) was targeted, 100 participants per subgroup were needed to obtain a 0.90 power to detect a 0.45 SD (“medium”) effect between groups with a 5% risk of type 1 error and a bidirectional two-sample homoscedastic *t* test [18,19]. To compensate for nonresponse and subgroup skewness, we intended to include an additional 300 participants per group, thereby totaling 12,000 participant invitations (400 per group and 30 groups) [20]. In order to anticipate the possibility of even lower participation, we obtained DHDA permission for sending up to 36,000 invitations in total (through up to three consecutive waves). Ultimately, two waves of surveys were required to achieve the necessary sample size.

We present RRs as counts and proportions, with stratification into 5-year age groups, survey wave groups (the person was part of which of the two survey waves), and municipality types (four categories) [21]. We compared the proportions between the groups by logistic regression (both unadjusted and mutually adjusted) and compared the participants with the general population by logistic regression with weights according to population size, using data from Statistics Denmark [22]. Comprehensive digital mailbox systems like the Danish system are not in place in all countries. Therefore, to get an impression of the general representativeness of our sample, we compared responders to all nonresponders in “A comparisons” without consideration for their opportunity to respond (having the digital mailbox). In “B comparisons,” we made comparisons between responders and nonresponders among those having the digital mailbox only in order to accentuate any active decision on whether to participate. Conducting both comparisons in parallel is necessary to allow for taking into account digital mailbox noncoverage and to rule out that we only obtained responses from a highly selected group of the population through our web-survey solution. Odds ratios (ORs) denote the probability of response (ie, OR >1 means more likely to respond). Analyses were carried out using Stata version 15.0 (Stata Corp LP).

### Ethics Approval and Consent to Participate

When conducting web-based surveys, ethical issues may arise with regard to procuring valid informed consent and respecting responders’ privacy [7]. The Regional Scientific Ethics Committee for Southern Denmark evaluated the project and concluded that the project could be implemented without their permission (case number 20182000-99). However, after ensuring that data management was in compliance with EU regulation 2016/679 and Directive 95/46/EC, General Data Protection Regulation, we obtained Data Protection Agency authorization through the regional municipality and DHDA permission to conduct the survey (number FSEID-00003692). Invitation letters to potential participants explained the purpose of the study and were distributed with a link to the questionnaire. Considering a person’s privilege to decline participation in our study, in addition to the introductory invitation letter, we considered that it was appropriate to send only one reminder after 14 days to augment the RR.

## Results

**Figure 1:**
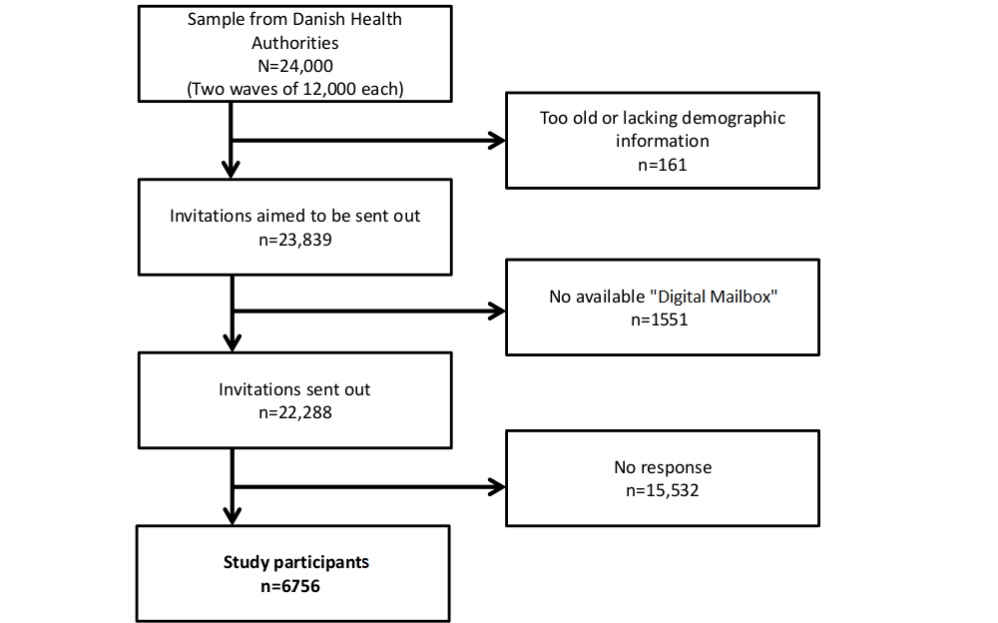
Flowchart showing the inclusion of study participants.

Participants were invited on January 24, 2019, and one reminder was sent after 14 days. To obtain a satisfactory sample size (n=100) in all (n=30) questionnaire variants, two consecutive waves of invitations were necessary. The second wave of invitations was sent out on March 07, 2019, with a reminder after 14 days. We thereby obtained a sample of 6756 participants. The flow chart is presented in Figure 1, and sample characteristics are presented in Table 1.

Among 161 excluded individuals, 117 were excluded because they either were too old or had unknown age according to register information from DHDA, 6 were excluded as they explicitly asked to be deleted from the research project, and 38 were excluded because they were too old when they responded to the survey. The overall RR was 30.31% (6756/22,288) among digital mailbox invitations and 28.34% (6756/23,839) in the general population. It appears from our study that the digital mailbox covered 93.49% (22,288/23,839) of our target population.

**Table 1:** Sample characteristics.

Characteristic		Responders^a^ (N=6756), median (IQR) or n		Nonresponders (N=15,532), median (IQR) or n		No digital mailbox (N=1551), median (IQR) or n	
**Age (years)**		59 (53-65)		55 (50-62)		60 (54-66)	
	45-49 (N=4721)		935		3633		153
	50-54 (N=5256)		1225		3751		280
	55-59 (N=4804)		1368		3147		289
	60-64 (N=4276)		1406		2515		355
	65-70 (N=4782)		1822		2486		474
**Municipality type**							
	Urban municipality (N=10,564)		2828		7049		687
	Rural municipality (N=6962)		2042		4443		477
	Urban-rural municipality (N=3947)		1183		2525		239
	Outskirts municipality (N=2366)		703		1515		148
**Invitation wave**							
	Wave 1 (N=11,869)		3395		7716		758
	Wave 2 (N=11,970)		3361		7816		793

^a^Responder numbers and response rates regarding the 30 different questionnaire variants of the survey are shown in Multimedia Appendix 1.

In Tables 2 and 3, responders and nonresponders are compared. It appeared that older men and men from rural areas had higher RRs.

**Table 2:** Comparison of responders and nonresponders according to age and dwelling.

Characteristic		Comparison A^a^ (N=23,839), OR (95% CI)^b^	Comparison A, *P* value	Comparison B^c^ (N=22,288), OR (95% CI)^b^		Comparison B, *P* value		
**Age (years)**			<.001			<.001		
	45-49	1 (reference)	N/A^d^		1 (reference)		N/A	
	50-54	1.12 (1.18-1.35)	<.001		1.27 (1.15-1.40)		<.001	
	55-59	1.61 (1.47-1.77)	<.001		1.69 (1.53-1.86)		<.001	
	60-64	1.98 (1.80-2.18)	<.001		2.17 (1.97-2.39)		<.001	
	65-70	2.49 (2.27-2.73)	<.001		2.85 (2.59-3.13)		<.001	
**Municipality type**			<.001			<.001		
	Urban municipality	1 (reference)	N/A		1 (reference)		N/A	
	Rural municipality	1.14 (1.06-1.21)	<.001		1.15 (1.07-1.23)		<.001	
	Urban-rural municipality	1.17 (1.08-1.27)	<.001		1.17 (1.08-1.27)		<.001	
	Outskirts municipality	1.16 (1.05-1.28)	.004		1.16 (1.05-1.28)		.004	
**Invitation wave**			N/A			N/A		
	Wave 1	1 (reference)	N/A		1 (reference)		N/A	
	Wave 2	1.03 (0.97-1.09)	.37		1.02 (0.97-1.08)		.43	

^a^Comparison A compares responders to all nonresponders with or without the digital mailbox.

^b^Odds ratios (ORs) denote the probability of response (ie, OR >1 means more likely to respond).

^c^Comparison B compares responders to nonresponders with the digital mailbox.

^d^N/A: not applicable.

**Table 3:** Comparison of responders and nonresponders according to age and dwelling adjusted for age, municipality, and wave.

Characteristic			Comparison A^a^ (N=23,839), OR (95% CI)^b^		Comparison A, *P* value		Comparison B^c^ (N=22,288), OR (95% CI)^b^		Comparison B, *P* value
**Age (years)**					N/A^d^				N/A
	45-49	1 (reference)		N/A		1 (reference)		N/A	
	50-54	1.23 (1.12-1.35)		<.001		1.27 (1.15-1.40)		<.001	
	55-59	1.61 (1.46-1.77)		<.001		1.69 (1.53-1.86)		<.001	
	60-64	1.97 (1.79-2.17)		<.001		2.16 (1.96-2.38)		<.001	
	65-70	2.48 (2.26-2.72)		<.001		2.83 (2.58-3.11)		<.001	
**Municipality type**					N/A				N/A
	Urban municipality	1 (reference)		N/A		1 (reference)		N/A	
	Rural municipality	1.10 (1.03-1.18)		.006		1.10 (1.03-1.18)		.005	
	Urban-rural municipality	1.16 (1.07-1.26)		<.001		1.15 (1.06-1.25)		.001	
	Outskirts municipality	1.10 (0.99-1.21)		.07		1.08 (0.98-1.20)		.12	
**Invitation wave**					N/A				N/A
	Wave 1	1 (reference)		N/A		1 (reference)		N/A	
	Wave 2	1.02 (0.96-1.08)		.55		1.02 (0.96-1.08)		.59	

^a^Comparison A compares responders to all nonresponders with or without the digital mailbox.

^b^Odds ratios (ORs) denote the probability of response (ie, OR >1 means more likely to respond).

^c^Comparison B compares responders to nonresponders with the digital mailbox.

^d^N/A: not applicable.

Table 4 illustrates the statistical differences between responders and nonresponders across questionnaire variants. One would expect a tendency toward lower RRs with increasing length of the questionnaire, that is, with higher group number from 1 through 10 and from main variant A through C. However, no clear association could be demonstrated.

As a proxy for the amount of resources required to complete the survey, we measured the time used by responders across variants (Multimedia Appendix 2). It is worth noting that no relevant difference could be established regarding response time across questionnaire variants. Correspondingly, when looking at RRs, no clear relevant association appeared between questionnaire variant and RR. Although “Group 10” had a notably lower RR than did the other groups, “Group 9” that had a similar length demonstrated no decrease in the RR.

Table 5 compares responders with the entire Danish population of men aged 45 to 70 years. It appears that compared with the general population, younger men and urban men are underrepresented in the sample.

**Table 4:** Comparison of responders and nonresponders regarding questionnaire variants.

Variants		Comparison B^a^ (N=22,288), OR (95% CI)^b^		Comparison B, *P* value		Adjusted comparison B^a^ (N=22,288), OR (95% CI)^b,c^		Adjusted comparison B, *P* value	
Randomization and vignette type				.004				N/A^d^	
**Number and vignette characteristics**				.02				N/A	
	**No patient participation^e^**								
	1		1 (reference)		N/A		1 (reference)		N/A
	2		1.10 (0.97-1.25)		.13		1.10 (0.97-1.25)		.15
	**Various means of informing patient and patient participation in decision making^f^**								
	3		1.07 (0.94-1.21)		.32		1.06 (0.93-1.21)		.36
	4		1.03 (0.91-1.17)		.62		1.04 (0.91-1.19)		.55
	5		1.02 (0.90-1.16)		.77		1.01 (0.89-1.15)		.85
	6		1.00 (0.88-1.13)		.97		0.99 (0.87-1.13)		.87
	7		0.97 (0.85-1.11)		.66		0.96 (0.84-1.10)		.56
	8		0.99 (0.87-1.12)		.85		0.99 (0.87-1.12)		.84
	**Patient involvement through shared decision making and use of a decision aid^g^**								
	9		1.03 (0.90-1.17)		.70		1.02 (0.90-1.16)		.75
	10		0.85 (0.75-0.97)		.02		0.85 (0.74-0.96)		.01
**Alternative and course**				.006				N/A	
	A: No cancer detected		1 (reference)		N/A		1 (reference)		N/A
	B: Treatable PCa^h^		1.01 (0.94-1.08)		.79		1.01 (0.94-1.09)		.72
	C: Fatal PCa		0.91 (0.85-0.98)		.008		0.91 (0.84-0.97)		.007

^a^Comparison B compares responders to nonresponders among all individuals with the digital mailbox.

^b^Odds ratios (ORs) denote the probability of response (ie, OR >1 means more likely to respond).

^c^Adjusted for age and geography and mutually adjusted.

^d^N/A: not applicable.

^e^For example, in one version of the vignette, the fictional doctor performs the prostate-specific antigen (PSA) test without any test information and the patient described in the vignette is later successfully treated for prostate cancer (PCa) (alternative B).

^f^For example, in one version, the patient chooses to have a PSA test following brief information about the test (showing no PCa; alternative A), and in other versions, the patient chooses not to have a test after being slightly nudged in favor or in disfavor of the PSA test and is later diagnosed with nontreatable PCa (alternative C).

^g^For example, in one version, the patient is subject to a shared decision-making aid and dialogue with the doctor, chooses not to take the PSA test, and is later diagnosed with treatable PCa (alternative B).

^h^PCa: prostate cancer.

**Table 5:** Comparison of responders with the entire Danish population of men.

Characteristic		Sample (all responders) (N=6756), n (%)	Denmark (N=951,247)^a^, n (%)	*P* value	*P* value adjusted^b^
**Age (years)**				<.001	<.001
	45-49	935 (13.84%)	195,838 (20.59%)		
	50-54	1225 (18.13%)	212,293 (22.32%)		
	55-59	1368 (20.25%)	188,671 (19.83%)		
	60-64	1,406 (20.81%)	169,672 (17.84%)		
	65-70	1822 (26.97%)	187,926 (19.76%)		
**Municipality type**				<.001	.006
	Urban municipality	2828 (41.86%)	425,073 (44.69%)		
	Rural municipality	2042 (30.22%)	279,102 (29.34%)		
	Urban-rural municipality	1183 (17.51%)	159,240 (16.74%)		
	Outskirts municipality	703 (10.41%)	90,985 (9.57%)		

^a^Danish men aged 45 to 70 years as of January 1, 2019.

^b^Adjusted for age and municipality type.

Tables 6 and 7 compare municipality-level characteristics in responders and nonresponders. In unadjusted models, higher population density, higher tax base, higher proportion of educated citizens, and higher proportion of citizens from non-Western countries were associated with lower RRs. Mutual adjustments were made; however, the associations weakened. Most consistently, RRs were found to be lower in population dense and higher tax base areas. Counts and frequencies corresponding to the data in Tables 6 and 7 are presented in Multimedia Appendix 3.

**Table 6:** Comparison of municipality-level characteristics in responders and nonresponders.

Characteristic		Comparison A^a^ (N=23,839), OR^b^ (95% CI)	Comparison A, *P* value	Comparison B^c^ (N=22,288), OR (95% CI)	Comparison B, *P* value
**Population density (citizens/km^2^)**					
	First tertile (least population density)	1 (reference)	N/A^d^	1 (reference)	N/A
	Second tertile (middle population density)	1.01 (0.95-1.08)	.75	1.01 (0.94-1.08)	.81
	Third tertile (most population density)	0.84 (0.79-0.90)	<.001	0.85 (0.79-0.91)	<.001
**Tax per citizen**					
	First tertile (lowest tax base)	1 (reference)	N/A	1 (reference)	N/A
	Second tertile (middle tax base)	1.00 (0.94-1.07)	.94	0.97 (0.93-1.07)	.91
	Third tertile (highest tax base)	0.86 (0.80-0.92)	<.001	0.85 (0.79-0.91)	<.001
**Proportion of citizens aged 25-64 years with higher education**					
	First tertile (fewest with high education)	1 (reference)	N/A	1 (reference)	N/A
	Second tertile (middle proportion with high education)	1.02 (0.96-1.09)	.52	1.02 (0.95-1.09)	.64
	Third tertile (most with high education)	0.89 (0.83-0.95)	.001	0.89 (0.83-0.95)	.001
**Number of citizens from non-Western countries per 10,000 people**					
	First tertile (fewest non-Western)	1 (reference)	N/A	1 (reference)	N/A
	Second tertile (middle number non-Western)	1.05 (0.98-1.12)	.15	1.07 (1.00-1.15)	.048
	Third tertile (most non-Western)	0.89 (0.83-0.95)	.001	0.91 (0.84-0.97)	.007

^a^Comparison A compares responders to all nonresponders with or without the digital mailbox.

^b^OR: odds ratio.

^c^Comparison B compares responders to nonresponders with the digital mailbox.

^d^N/A: not applicable.

**Table 7:** Comparison of municipality-level characteristics adjusted for population density, tax per citizen, proportion with higher education, and number of citizens from non-Western countries.

Characteristic		Comparison A^a^ (N=23,839), OR^b^ (95% CI)	Comparison A, *P* value	Comparison B^c^ (N=22,288), OR (95% CI)	Comparison B, *P* value
				
**Population density (citizens/km^2^)**					
	First tertile (least population density)	1 (reference)	N/A^d^	1 (reference)	N/A
	Second tertile (middle population density)	0.98 (0.90-1.08)	.71	0.98 (0.89-1.07)	.62
	Third tertile (most population density)	0.85 (0.73-1.00)	.049	0.84 (0.71-0.99)	.03
**Tax per citizen**					
	First tertile (lowest tax base)	1 (reference)	N/A	1 (reference)	N/A
	Second tertile (middle tax base)	0.98 (0.91-1.06)	.58	0.97 (0.90-1.05)	.45
	Third tertile (highest tax base)	0.91 (0.83-1.00)	.054	0.89 (0.81-0.98)	.02
**Proportion of citizens aged 25-64 years with higher education**					
	First tertile (fewest with high education)	1 (reference)	N/A	1 (reference)	N/A
	Second tertile (middle proportion with high education)	1.07 (0.98-1.18)	.14	1.07 (0.98-1.18)	.15
	Third tertile (most with high education)	1.03 (0.92-1.16)	.59	1.03 (0.92-1.16)	.57
**Number of citizens from non-Western countries per 10,000 people**					
	First tertile (fewest non-Western)	1 (reference)	N/A	1 (reference)	N/A
	Second tertile (middle number non-Western)	1.05 (0.98-1.13)	.18	1.08 (1.00-1.16)	.052
	Third tertile (most non-Western)	1.04 (0.92-1.19)	.50	1.08 (0.95-1.23)	.22

^a^Comparison A compares responders to all nonresponders with or without the digital mailbox.

^b^OR: odds ratio.

^c^Comparison B compares responders to nonresponders with the digital mailbox.

^d^N/A: not applicable.

## Discussion

### Principal Findings

Using web-based surveys to collect data for public health research provides an opportunity to get easy access to potential responders while reducing efforts and research costs [7]. As is the case with other types of surveys, however, responders sometimes may not accurately represent the group of interest. In this study, we report on the representativeness of a large sample of adult men recruited through the use of a national web-based communication channel (the Danish digital mailbox). Our findings do not indicate that we received responses from only a highly selected group of the population. Nevertheless, we found that older men and men living in rural areas were more likely to respond than younger men, while RRs were lower in high economic resource areas. We discuss the findings in detail with reference to the existing literature below.

### Comments Regarding the Use of Digital Mailbox Solutions for Research

There are multiple benefits of using web surveys for health research [9,23]. For example, participants may enter their responses confidentially and directly into the electronic database, allowing for more complete responses to sensitive questions and making subsequent data management much easier [23,24]. Furthermore, web surveys benefit from the ability to automatically branch into different scenarios, skipping irrelevant questions, etc [24]. As a result, savings are potentially larger compared with traditional mail or telephone surveys [25]. Uneven distribution in the use of information technologies among groups with different backgrounds may, however, challenge the use of web-based surveys for research [7]. In this regard, distributing surveys through a public platform, which is essentially mandatory for citizens to use, may be an attractive solution. Danish public authorities like hospitals and municipalities use the digital mailbox to inform citizens about tax issues, medical examinations, etc, adding to its legitimacy as an important platform for information exchange [9,26]. This may itself promote survey participation and, in part, prevent nonresponse, if not misused. It is noteworthy that 93.49% (22,288/23,839) of our target population was registered with the digital mailbox, a finding that corresponds very well with available public reports on digital mailbox coverage (92.9% among all Danish men as of the first quarter of 2019 [27]). As discussed below, this relatively high coverage is not necessarily reflected in a high RR.

The digital mailbox has been previously used for national-level survey research. Researchers recently reported on their experiences with using the digital mailbox for distribution of “The Danish Health and Morbidity Surveys” to describe the status and trends in health and morbidity in the adult Danish population aged 16 years or older [9]. The authors found that in 2017, 90.1% of their sample was registered to use the digital mailbox; however, there were variations across age groups from 98.7% among individuals aged 24 years to 68.7% among individuals aged 65 years. Among men aged 45 years, they found RRs between 57.1% and 66.3%. Their survey may generally appeal to a broader audience thereby contributing to a higher RR. The web-based survey methodology constitutes a particularly attractive opportunity for sending reminders with very little effort, and “The Danish Health and Morbidity Surveys” indeed benefitted from this ability by using a total of four reminders plus the introduction letter to increase the RR. Using multiple reminders, however, could potentially raise ethical concerns with regard to respecting citizens’ rights not to join a research project.

### Discussion Regarding the Response Rate and Representativeness

In “Encyclopedia of Survey Research Methods,” Davern concluded that the standards for true representativeness in surveys are rarely met; however, “the biases produced by failures often are not severe enough to threaten the ultimate value of the survey findings” [8]. Likewise, there is no clear answer to the question about when an RR is acceptable [25]. Based on a review of 30 published studies, the RR itself was concluded to not be a good indicator of the magnitude of nonresponse bias [25,28]. Although very high RRs tended to reduce the chance of nonresponse bias, when bias did occur, the degree of bias was not necessarily low [28]. Furthermore, considerable variation often appeared in the degree of bias among variables within a study.

The RR generally can be affected by many factors [29,30]. For example, a smaller proportion of individuals responding probably would be expected in a more time-consuming survey. Previous research on the relationship between survey length and the RR has predominantly focused on other survey modalities (postal service and phone interviews) [31]. Our findings suggest that the relationship between the amount of survey material and the RR may not be that clear for web-based surveys. In any case, our RR (30%) was lower than that usually seen in Danish population-based surveys [32,33]. In addition to the impact of only sending one reminder, the smaller RR may mirror the fact that the questionnaire was not available as a hard copy. Additionally, it may reflect that the RR is sometimes lower among men than among women [7,33]. Furthermore, it should be kept in mind that there was no direct benefit for survey responders apart from the sense of contributing to scientific knowledge about a patient’s wish for involvement in health care decisions. Finally, to solve the problem with missing values, we constructed the web survey in such a way that participation without fully completing the questionnaire was not possible. This may have contributed to the smaller RR.

Our finding that RRs are higher in older men conflicts with the finding in a previous Danish survey [9] but seems to agree with the finding in another Danish investigation [33]. Keeping in mind that PCa risk rises with age, it is understandable that older men will be more concerned about this issue [12]. The higher RR may, however, also reflect that even if younger citizens have better access to the internet, older and retired people have more time available for research participation [7]. In accordance with previous findings, the RR seemed smaller in high income populations [33]. Associations overall were rather similar between comparisons of responders to all nonresponders and comparisons of responders to nonresponders with the digital mailbox, suggesting that nonresponders without the digital mailbox were fairly similar to nonresponders with the digital mailbox in terms of the variables under study.

### Interpretation of the Study Findings

Our findings make it necessary to consider the possible consequences for further analyses of our survey data and the question of whether the composition of our sample tends to overrepresent particular viewpoints on health care decision-making. Regarding the association between age and preferences for patient involvement, some previous studies pointed toward older people having preferences for less participation in decision-making [34-37]. On the other hand, in the review by Hubbard et al that included 11 studies on the association between age and role preferences in decisions about cancer treatment, no general conclusion could be drawn [38]. Five studies reported that younger people were more likely to prefer a collaborative or active role in decision-making, but regarding PCa in particular, no relevant association with responder age was found in any one of three studies*.*

We found the RR to be much higher in rural municipalities. Furthermore, the RR was lower in areas with a higher tax base. Although the RR initially seemed to be lower in higher education areas, this association faded in adjusted analyses. Additionally, the RR seemed to be lower in areas with a higher proportion of citizens from non-Western countries, which might partly reflect the fact that the questionnaire was only available in Danish, although this association also disappeared in adjusted analyses controlling for the association with municipality-level population density and average tax per citizen.

A Cochrane review on patient decision aids suggested that the desire for involvement in health care decision-making, rather than being a stable trait, should be considered an adaptable way of thinking (“state”) [39]. People simply have to be informed about why they should participate in a certain decision and understand the importance of their own preferences for outcomes of options (which outcomes matter most to them) before they are asked about their desire to participate. In spite of this, some studies have investigated associations between sociodemographic factors and preferences for involvement in decisions. Degner and Sloan found a nonrelevant trend for individuals from more rural areas to give away control to their physicians to a greater extent [36]. With regard to the impact of education, in the study by Rovner et al, college-educated men tended to want to make their own decisions about health care in benign prostatic hyperplasia, whereas noncollege-educated men tended to desire a shared approach [40]. Additionally, those preferring that decisions should be made mainly by the patient had a higher income. Correspondingly, in a large survey, Levinson et al found that more educated and healthier people were more likely to prefer an active role in decision-making [41]. On the other hand, African-American and Hispanic responders were more likely to prefer a passive role in decision-making. Generally, preferences for involvement increased with age up to 45 years and then declined. In a later study, Peek et al confirmed no racial differences in preferences for participation in decision-making [42].

While previous studies suggest that preferences for participation in health care decision-making may vary with the aforementioned factors, the latter factors may play no clear-cut role when it comes to decisions about cancer treatment. Hence, it was generally concluded from the review by Hubbard et al of 31 papers in total investigating patient preferences for participation in decision-making that the evidence on associations with age as well as gender, education level, marital status, socioeconomic status, and health status was inconclusive [38]. However, sample sizes were small in the majority of the studies. Patient preferences for involvement in decision-making are hypothesized to vary with age, socioeconomic status, etc [37], and therefore, statistical weighting may be warranted to control for skewness.

### Limitations

Some previous studies have suggested gender differences regarding preferences for participation in decision-making [34-36,41,43]. This hardly can be controlled for in a survey including only male responders, but points to possible limitations regarding the generalizability of survey RR findings to women. In this regard, however, the role of gender has been questioned, at least when it comes to cancer issues [38]. Furthermore, it should be noted that only municipality-level register data on responders’ sociodemographic characteristics were used. For example, the income of individual responders was not available. Likewise, more complex issues with regard to, for example, psychological disposition was not taken into consideration. In this regard, preferences for involvement in decision-making actually have been hypothesized to vary among personality types, while individuals’ likelihood of survey participation simultaneously may vary with personality style [44-46].

### Conclusion

The generalizability of survey findings to target populations is an important goal in research using web survey methodology. We wanted to establish the representativeness of responders in a large national survey investigating the desire of men to participate in decision-making about undergoing a PSA test, using the national digital mailbox platform. Our comparisons of responders to nonresponders point toward a reasonable representativeness of the sample. With regard to the result that responders did not fully represent men aged 45 to 70 years, previous research findings suggest that the variation found in our sample may not necessarily deteriorate forthcoming analyses on preferences for involvement in decision-making. In any case, identification of imbalances allows for statistical corrections to be made during the analysis of survey responses.

## Appendix

Multimedia Appendix 1 Respondent numbers and response rates regarding the different questionnaire scenario variants of the survey.

Multimedia Appendix 2 Time spent answering the questionnaire.

Multimedia Appendix 3 Counts and proportions for comparisons of municipality-level characteristics in respondents and nonrespondents.

## Abbreviations

DHDA: Danish Health Data Authority
OR: odds ratio
PCa: prostate cancer
PSA: prostate-specific antigen
RR: response rate
STROBE: Strengthening the Reporting of Observational Studies in Epidemiology
