# Supplementary material for: Sociodemographic Representativeness in a Nationwide Web-Based Survey of the View of Men on Involvement in Health Care Decision-Making: Cross-Sectional Questionnaire Study
Source: J Med Internet Res. 2020 Sep 2;22(9):e19517. doi: 10.2196/19517 (PMC7495257; doi:10.2196/19517)
Supplement: Multimedia Appendix 1 [file jmir_v22i9e19517_app1.docx]

**Appendix 1: Respondent numbers and response rates regarding the different questionnaire scenario variants of the survey**

| **Questionnaire variant^a^** | Participants (N=6,756) | No response (N=15,532) | No Digital Mailbox(N=1,551) |
| --- | --- | --- | --- |
| 1a | 238 (30.1%) | 500 (63.1%) | 54 (6.8%) |
| 1b | 222 (28.0%) | 514 (64.9%) | 56 (7.1%) |
| 1c | 209 (26.3%) | 531 (66.7%) | 56 (7.0%) |
| 2a | 267 (33.6%) | 482 (60.6%) | 46 (5.8%) |
| 2b | 244 (30.7%) | 492 (61.9%) | 59 (7.4%) |
| 2c | 211 (26.5%) | 536 (67.4%) | 48 (6.0%) |
| 3a | 228 (28.7%) | 520 (65.4%) | 47 (5.9%) |
| 3b | 243 (30.6%) | 493 (62.0%) | 59 (7.4%) |
| 3c | 230 (29.0%) | 505 (63.6%) | 59 (7.4%) |
| 4a | 238 (30.0%) | 508 (64.1%) | 47 (5.9%) |
| 4b | 230 (29.0%) | 497 (62.6%) | 67 (8.4%) |
| 4c | 217 (27.3%) | 526 (66.2%) | 51 (6.4%) |
| 5a | 228 (28.7%) | 527 (66.4%) | 39 (4.9%) |
| 5b | 229 (28.8%) | 519 (65.3%) | 47 (5.9%) |
| 5c | 230 (29.0%) | 510 (64.2%) | 54 (6.8%) |
| 6a | 208 (26.2%) | 538 (67.7%) | 49 (6.2%) |
| 6b | 238 (30.0%) | 491 (61.8%) | 65 (8.2%) |
| 6c | 220 (27.7%) | 513 (64.6%) | 61 (7.7%) |
| 7a | 225 (28.3%) | 518 (65.1%) | 53 (6.7%) |
| 7b | 233 (29.3%) | 508 (63.9%) | 54 (6.8%) |
| 7c | 201 (25.3%) | 540 (68.0%) | 53 (6.7%) |
| 8a | 219 (27.6%) | 530 (66.8%) | 44 (5.5%) |
| 8b | 230 (29.1%) | 515 (65.1%) | 46 (5.8%) |
| 8c | 222 (27.9%) | 524 (65.9%) | 49 (6.2%) |
| 9a | 261 (32.8%) | 487 (61.2%) | 48 (6.0%) |
| 9b | 221 (27.7%) | 535 (67.1%) | 41 (5.1%) |
| 9c | 210 (26.4%) | 536 (67.4%) | 49 (6.2%) |
| 10a | 196 (24.6%) | 549 (68.9%) | 52 (6.5%) |
| 10b | 214 (26.8%) | 538 (67.4%) | 46 (5.8%) |
| 10c | 194 (24.4%) | 550 (69.1%) | 52 (6.5%) |

^a^ Scenarios have an identical core structure though differ regarding the degree of patient involvement and the decision to have a PSA or not from no involvement in version 1 to involvement through shared decision-making in 10, and regarding outcome from course with no prostate cancer in ‘a’ to non-treatable prostate cancer in ‘c’.
