# Supplementary material for: Sociodemographic Representativeness in a Nationwide Web-Based Survey of the View of Men on Involvement in Health Care Decision-Making: Cross-Sectional Questionnaire Study
Source: J Med Internet Res. 2020 Sep 2;22(9):e19517. doi: 10.2196/19517 (PMC7495257; doi:10.2196/19517)
Supplement: Multimedia Appendix 2 [file jmir_v22i9e19517_app2.docx]

**Appendix 2:** Time spent answering the questionnaire

| **Questionnaire variant** | Number of answers included^a^ | Mean (sd) in minutes^b^ |
| --- | --- | --- |
| 1a | 179 | 20.2 (95.7) |
| 1b | 153 | 18.2 (111.7) |
| 1c | 153 | 64.1 (500.0) |
| 2a | 189 | 9.4 (13.4) |
| 2b | 178 | 9.4 (7.9) |
| 2c | 148 | 9.8 (5.8) |
| 3a | 165 | 8.5 (43.7) |
| 3b | 178 | 11.1 (13.4) |
| 3c | 168 | 5.4 (40.0) |
| 4a | 165 | 7.0 (33.0) |
| 4b | 168 | 12.7 (42.3) |
| 4c | 169 | 8.1 (26.8) |
| 5a | 171 | 11.2 (43.6) |
| 5b | 166 | 19.0 (93.0) |
| 5c | 174 | 10.1 (7.7) |
| 6a | 153 | 9.0 (3.7) |
| 6b | 158 | 19.8 (123.8) |
| 6c | 158 | 7.4 (48.5) |
| 7a | 165 | 7.0 (28.7) |
| 7b | 176 | 3.1 (47.0) |
| 7c | 146 | 12.0 (62.7) |
| 8a | 167 | 12.9 (47.2) |
| 8b | 169 | 36.7 (342.6) |
| 8c | 167 | 8.4 (54.4) |
| 9a | 198 | 31.9 (308.9) |
| 9b | 157 | 12.1 (14.9) |
| 9c | 150 | 15.8 (30.7) |
| 10a | 144 | 10.3 (6.1) |
| 10b | 166 | 14.2 (45.4) |
| 10c | 137 | 14.1 (26.8) |
| 1 | 485 | 33.4 (293.7) |
| 2 | 515 | 9.5 (9.9) |
| 3 | 511 | 8.4 (34.8) |
| 4 | 502 | 9.3 (34.6) |
| 5 | 511 | 13.4 (58.9) |
| 6 | 469 | 12.1 (77.2) |
| 7 | 487 | 7.1 (47.5) |
| 8 | 503 | 19.4 (202.8) |
| 9 | 505 | 21.0 (194.2) |
| 10 | 447 | 13.0 (31.6) |
| a | 1696 | 13.2 (113.7) |
| b | 1669 | 15.5 (126.4) |
| c | 1570 | 15.2 (160.6) |

^a^ Variants do not summarize to 6,756 because of missing information regarding time spend answering the questionnaire

^b^ P-values for difference in time spend answering: All 30 groups: P=0.175, Groups 1-10: P=0.074

Groups a-c: P=0.861
