# Supplementary material for: Sociodemographic Representativeness in a Nationwide Web-Based Survey of the View of Men on Involvement in Health Care Decision-Making: Cross-Sectional Questionnaire Study
Source: J Med Internet Res. 2020 Sep 2;22(9):e19517. doi: 10.2196/19517 (PMC7495257; doi:10.2196/19517)
Supplement: Multimedia Appendix 3 [file jmir_v22i9e19517_app3.docx]

**Appendix 3: Counts and proportions of comparison of municipality level characteristics in respondents and non-respondents**

|  | **Respondents**^a^  **N=6,756** | **No response**  **N=15,532** | **{No Digital Mailbox N=1,551** |
| --- | --- | --- | --- |
|  |  |  |  |
| **Population density (citizens/km^2^)** |  |  |  |
|  |  |  |  |
| 1. Tertile (least pop. dense) | 2347 (29.3%) | 5138 (64.2%) | 513 (6.4%) |
| 2. Tertile (middle pop. dense) | 2441 (29.6%) | 5299 (64.2%) | 513 (6.2%) |
| 3. Tertile (most pop. dense) | 1968 (25.9%) | 5095 (67.1%) | 525 (6.9%) |
|  |  |  |  |
| **Tax per citizen** |  |  |  |
|  |  |  |  |
| 1. Tertile (lowest tax base) | 2381 (29.2%) | 5207 (63.9%) | 555 (6.8%) |
| 2. Tertile (middle tax base) | 2459 (29.3%) | 5398 (64.3%) | 537 (6.4%) |
| 3. Tertile (highest tax base) | 1916 (26.2%) | 4927 (67.5%) | 459 (6.3%) |
|  |  |  |  |
| **Proportion citizens aged 25-64-years with higher education** |  |  |  |
|  |  |  |  |
| 1. Tertile (fewest with high educ.) | 2306 (29.0%) | 5128 (64.5%) | 522 (6.6%) |
| 2. Tertile (middle prop. high educ.) | 2362 (29.4%) | 5167 (64.4%) | 493 (6.1%) |
| 3. Tertile (most with high educ.) | 2088 (26.6%) | 5237 (66.6%) | 536 (6.8%) |
|  |  |  |  |
| **Amount of citizens from non-western countries per 10,000** |  |  |  |
|  |  |  |  |
| 1. Tertile (fewest non-western) | 2293 (28.8%) | 5235 (65.7%) | 446 (5.6%) |
| 2. Tertile (middle amount non-west.) | 2426 (29.8%) | 5167 (63.4%) | 551 (6.8%) |
| 3. Tertile (most non-western) | 2037 (26.4%) | 5130 (66.4%) | 554 (7.2%) |
